# Die Abdominal Pain Unit als Behandlungspfad: Strukturierte Versorgung von Patient*innen mit atraumatischen Bauchschmerzen in der Notaufnahme

**DOI:** 10.1007/s00063-021-00887-0

**Published:** 2021-12-20

**Authors:** Lukas Helbig, Britta Stier, Claudia Römer, Maik Kilian, Anna Slagman, Angelika Behrens, Vera Stiehr, Jörn Ole Vollert, Ulrike Bachmann, Martin Möckel

**Affiliations:** 1 Akut- und Notfallmedizin, Charité – Universitätsmedizin Berlin, Charitéplatz 1, 10117 Berlin, Deutschland; 2 Abteilung für Allgemein- und Viszeralchirurgie, Evangelische Elisabeth Klinik Berlin, Lützowstraße 26, 10785 Berlin, Deutschland; 3 Abteilung für Innere Medizin, Gastroenterologie und Pneumologie, Evangelische Elisabeth Klinik Berlin, Lützowstraße 26, 10785 Berlin, Deutschland

**Keywords:** Behandlungsprozedere, Standardisierung, Akutversorgung, Effizienz, APU, Treatment procedure, Standardization, Acute care, Efficiency, APU

## Abstract

**Hintergrund:**

Patient*innen mit atraumatischen Bauchschmerzen (aBS) sind in der Notaufnahme (NA) häufig und haben bei einem sehr weiten Spektrum verschiedener ursächlicher Diagnosen eine relativ hohe Krankenhaussterblichkeit. Eine schnelle, zielführende Diagnostik ist in diesem Zusammenhang essenziell.

**Methode:**

In einem Delphi-Verfahren mit Vertreter*innen verschiedener Fachrichtungen wurde ein diagnostischer Behandlungspfad entworfen, der als „Abdominal Pain Unit“ (APU) bezeichnet wird.

**Ergebnis:**

Der Behandlungspfad wurde als erweiterte Ereignisprozesskette dargestellt und die jeweiligen Entscheidungsfelder mit Dokumenten für ein standardisiertes Vorgehen hinterlegt.

**Diskussion:**

Der APU-Behandlungspfad etabliert eine konsistente Versorgungsstruktur für Bauchschmerzpatient*innen. Er hat das Potenzial die Versorgungsqualität zu verbessern und die intrahospitale Mortalität langfristig zu senken.

**Zusatzmaterial online:**

Die Onlineversion dieses Beitrags (10.1007/s00063-021-00887-0) enthält die Tabellen S1–S9.

## Abstract (en)

**Background:**

Patients with atraumatic abdominal pain are common in the emergency department and have a relatively high hospital mortality, with a very wide spectrum of different causes. Rapid, goal-directed diagnosis is essential in this context.

**Methods:**

In a Delphi process with representatives of different disciplines, a diagnostic treatment pathway was designed, which is called the Abdominal Pain Unit (APU).

**Results:**

The treatment pathway was designed as an extended event process chain. Crucial decision points were specified using standard operating procedures.

**Discussion:**

The APU treatment pathway establishes a consistent treatment structure for patients with atraumatic abdominal pain. It has the potential to improve the quality of care and reduce intrahospital mortality over the long term.

## Einleitung

Atraumatische Bauchschmerzen (aBS) stellen ein häufiges Problem in deutschen Notaufnahmen dar und sind, bei einer hohen Heterogenität möglicher Ursachen, mit einer relativ hohen Krankenhaussterblichkeit vergesellschaftet [3, 19]. Häufig ist die Versorgung von Patient*innen mit Bauchschmerzen von der Erfahrung und Intuition des ärztlichen Personals abhängig. Analog zur „Chest Pain Unit“ (CPU) wurde ein strukturierter Diagnostik- und Behandlungspfad entworfen, um die Versorgung dieser Patient*innen standardisiert und effizient zu gestalten: die Abdominal Pain Unit (APU).

Der atraumatische Bauchschmerz wird als nichttraumatisch bedingter Schmerz im Bauchraum, der nicht länger als 5–7 Tage andauert, definiert [3]. Ein klarer Therapiepfad ist aufgrund der Vielzahl und komplikationsreichen Differenzialdiagnosen bisher nicht eindeutig vorgegeben. Eine internationale, fachgesellschaftsübergreifende Leitlinie existiert aktuell nicht.

Der atraumatische Bauchschmerz ist eines der häufigsten Leitsymptome in der Notaufnahme (NA). Die Prävalenz liegt zwischen 5 und 20 % [1, 4] aller Notfälle und ist nach Trentzsch et al. der häufigste chirurgische Notfall [19]. Die Krankenhausmortalität von Patient*innen mit akuten Bauchschmerzen liegt mit 5,6 %, im Vergleich zum Leitsymptom Brustschmerz (0,9 %), dessen Versorgung dem Therapiepfad der Chest Pain Unit (CPU) mit kontinuierlichem Monitoring und Biomarkerbestimmung folgt, sehr hoch [13]. Aus der hohen Mortalität und der Heterogenität der zugrunde liegenden, teilweise sehr zeitkritischen Diagnosen ergibt sich die Notwendigkeit einer schnellen und zielführenden Diagnostik.

Nicht nur aufgrund des demographischen Wandels mit einem steigenden Anteil der älteren Bevölkerung [4, 17], sondern auch aufgrund des vermehrten Patientenaufkommens in den Notaufnahmen [10, 11] müssen die aktuellen Versorgungsstrukturen kritisch evaluiert werden. Die NA als intersektorale Schnittstelle muss stationären und ambulanten Qualitätsanforderungen gerecht werden, somit ist die einheitliche Qualitätsdokumentation und -messung in der Notfallmedizin anzustreben [8]. Durch die Einführung von neuen Versorgungs- und Abrechnungsformen, im Sinne einer APU, kann die Versorgungsqualität in Zukunft verbessert werden.

Das Ziel der APU ist es, die Behandlung von Patienten mit aBS in der NA zu standardisieren. Hierfür wurde ein Behandlungspfad entwickelt, der alle potenziellen Ursachen berücksichtigt und anhand der hinterlegten Standard Operating Procedures (SOP) vom ärztlichen Personal in der NA – unabhängig von dessen Ausbildungsstand – befolgt werden kann. Die Darstellung des Prozesses erfolgt zeitgemäß über eine Anwendungssoftware (App, entwickelt durch RealCore Group GmbH, Essen, Deutschland).

## Methoden

In einem mehrstufigen Delphi-Verfahren erfolgte in einem interdisziplinären Expert*innengremium der Fachgebiete Chirurgie, Epidemiologie, inneren Medizin, Prozessforschung und Notfallmedizin die Entwicklung des Diagnostik- und Behandlungspfads. Dabei waren 2 chirurgische, 3 internistische/notfallmedizinische Expert*innen sowie jeweils ein(e) Epidemiologie- und ein(e) Prozessexpert*in vertreten.

Im ersten Schritt wurde eine erweiterte ereignisgesteuerte Prozesskette (eEPK) für Patient*innen mit aBS in der NA entworfen. Das Gremium konsentierte zunächst den Ablauf der eEPK und arbeitete anschließend die zugehörigen SOP aus.

Im nächsten Schritt erfolgte die Visualisierung der eEPK analog der Publikationen von Möckel et al., Vollert et al. und Pöss et al. [12, 15, 16, 21]. Die eEPK stellt Diagnostik- und Therapieprozesse als strukturierten Patientenpfad dar. Aus eingetretenen Ereignissen (rot, Sechseck) resultieren Handlungen (grün, abgerundetes Rechteck), die durch SOP (gelb, Rechteck) ergänzt werden, um einen effizienten und sicheren Patientenpfad aufzubauen und zu konkretisieren. Die SOP können variiert und an lokale Bedürfnisse angepasst werden.

Zur detaillierten Erläuterung der eEPK wird auf Möckel et al. verwiesen [12, 21].

Neben den qualitativen Überlegungen, die in den ersten Schritten des Delphi-Prozesses zur Erstellung der eEPK führten, ist es essenziell, auch die Häufigkeit des Auftretens bestimmter Krankheitsbilder und dafür sinnvoller Diagnostik zu betrachten. Im finalen Schritt des Delphi-Verfahrens erfolgte daher, entlang der eEPK, eine prospektive Analyse von zuvor im Rahmen einer anderen Studie (CHARITEM-Studie [13]) erhobenen Routinedaten zu Patient*innen in Notaufnahmen der Charité. Die Ergebnisse dieser quantitativen Datenanalyse halfen dabei, besondere diagnostische Entscheidungspunkte zu identifizieren und diese im Diagnostikpfad der eEPK entsprechend zu berücksichtigen.

## Ergebnisse

### Charakteristika der Patient*innen mit atraumatischen Bauchschmerzen

Im Rahmen des Delphi-Verfahrens zur Erstellung der eEPK erfolgte eine Analyse der im Rahmen der CHARITEM-Studie [13] erhobenen Routinedaten von Patient*innen mit aBS.

Wir untersuchten 3824 Patient*innen mit aBS von insgesamt 34.333 NA-Patient*innen im Jahr 2011.

Einen Überblick der Patient*innencharakteristika und die häufigsten verschlüsselten Diagnosen liefern Tab. 1 und 2.

**Table 1:** 

	Alle Patient*innen *N* = 34.333	Bauchschmerzen als Hauptbeschwerde *N* = 3824 (11,1 %)
*Alter (Jahre, (Median 25/75))*	57 (38/71)	45 (30/62)
*Geschlecht (weiblich/männlich, in %)*	51,2/48,8	55,6/44,4
*Nationalität (%)*		
Deutsch	91,2	88,6
Türkisch	3,4	4,7
Andere europäische Länder	3,3	3,6
Nichteuropäische Länder	2,2	3,0
*Verbleib (%)*		
Ambulant	60,6	68,4
Stationär	39,4	31,6
*Intrahospitale Mortalität (%)*	4,7	5,1

**Table 2:** 

ICD-10	Diagnose	*n* (%)
K85	Akute Pankreatitis	113 (9,4)
K80	Cholelithiasis	87 (7,2)
K56	Paralytischer Ileus und intestinale Obstruktion ohne Hernie	84 (7,0)
K29	Gastritis und Duodenitis	59 (4,9)
K57	Divertikelkrankheit des Darms	52 (4,3)
K35	Akute Appendizitis	44 (3,6)
K83	Sonstige Krankheiten der Gallenwege	39 (3,2)
K50	Morbus Crohn (Enteritis regionalis)	33 (2,7)
A41	Sonstige Sepsis	31 (2,6)

### Der APU-Algorithmus

**Figure 1:**
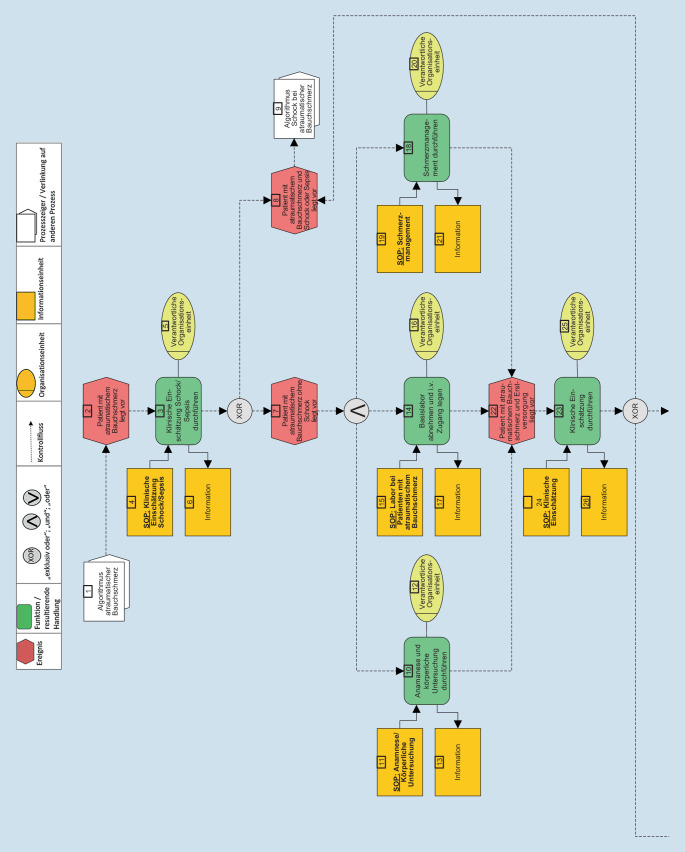

Die Abb. 1 stellt die eEPK zum Leitsymptom aBS als Ergebnis des Delphi-Verfahrens dar. Der Algorithmus beginnt, sobald ein triagierter Patient mit aBS in der NA vorstellig wird, d. h., es liegen bereits Vitalparameter und die Eingruppierung in eine Triagestufe vor. Die entsprechenden SOP finden sich in den Tab. 3 und 4 und im Onlinezusatzmaterial (Tabellen S1–S9).

**Figure 2:**
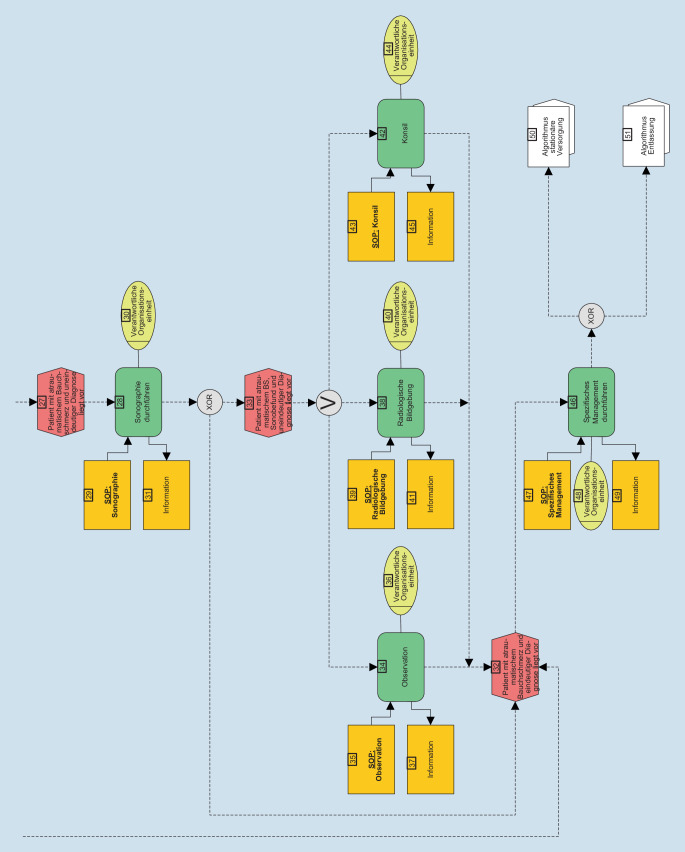

**Table 3:** 

SOP Klinische Einschätzung Schock/Sepsis	
Informationstext in der APP	Items zur Auswahl in der APP
*1. Vitalparameter (HF, RR, AF, S*_*a*_*O*_*2*_*) messen; Kriterien für Schock:*	*Atemfrequenz >* *22*
a. Systolischer Blutdruck (SBP) < 90 mm Hg	*GCS* *<* *15*
*2. In Einzelfällen normaler Blutdruck aber spontaner Abfall >* *30* *mm* *Hg bzw.*	Punkte werden von App errechnet
*3. Bei bereits laufender Katecholamintherapie*	*RR* *<* *100* *mm* *Hg*
*4. Hypoxie? Erhöhte Atemfrequenz?*	Bei ≥ 2 Punkten
*5. Vigilanz prüfen (GCS)*	Schock
*6. Temperatur messen (Fieber, Hypothermie)*	*Ja*
*7. Klinische Untersuchung durchführen Kriterien für Schock:*	Behandlungsende Datum/Uhrzeit Verlegung Schockraum/ITS
a. Kalte, marmorierte Extremitäten	Einleitung einer antibiotischen Therapie noch vor Ort
b. Zyanose	*Nein*
c. Kaltschweißigkeit	
d. Oligurie	
e. Ungewöhnliche Agitation	
*8. qSOFA bestimmen (AF* *≥* *22, GCS* *<* *15, RR systolisch* *≤* *100* *mm* *Hg)*	
*9. Bei Schock und/oder eindeutigen Zeichen der Sepsis (qSOFA* *≥* *2): Verlegungsziel ITS*	

*HF* Herzfrequenz, *RR* Blutdruck, *AF* Atemfrequenz, *S*_*a*_*O*_*2*_ Sauerstoffsättigung, *GCS* Glasgow Coma Scale, *qSOFA* quick Sequential Organ Failure Assessment, *ITS* Intensivstation

**Table 4:** 

SOP Klinische Einschätzung	
Informationstext aus der APP	
1. **Dynamik des Krankheitsbilds**	*Beurteilen Sie, ob sich das Krankheitsbild in den letzten (≤* *6) Stunden verschlechtert, gebessert oder stabil geblieben ist, und dokumentieren Sie Ihr Ergebnis*
2. **Akuität des Krankheitsbilds**	*Beurteilen Sie, ob Sie das Krankheitsbild des Patienten als hochakut (sofortiger Handlungsbedarf), subakut (dringender Handlungsbedarf innerhalb <* *2* *h), stabil (zeitnahe Versorgung, ggf. ambulant) einstufen, und dokumentieren Sie Ihr Ergebnis*
3. **Hämodynamische Stabilität**	*Beurteilen Sie, ob der Patient hämodynamisch stabil ist. Dokumentieren Sie Ihr Ergebnis. Verlegen Sie den Patienten auf eine Intensivstation, wenn der Patient hämodynamisch instabil ist. Siehe dazu auch SOP Feld 4 (Schock & Sepsis)*
4. Formulieren Sie in Bezug auf die Anamnese, den klinischen Untersuchungsbefund und die erhobenen Untersuchungsbefunde **eine Verdachtsdiagnose** und dokumentieren Sie diese	*–*
5. „**Do not miss“**	*Beachten Sie immer folgende Differenzialdiagnosen:*
	Rupturiertes Bauchaortenaneurysma
	Inkarzerierte Hernie
	Hodentorsion
	Hohlorganperforation
	Ileus
	Mesenterialischämie
	Milzruptur
	Myokardinfarkt
	Bedenken Sie, dass bei sehr alten und sehr jungen Patient*innen Symptome unspezifisch sein können; beachten Sie Sprachbarriere und kulturelle Besonderheiten
6. Führen Sie großzügig eine Sonographie zur weiteren diagnostischen Einordnung durch. Falls Sie nicht **Facharzt **sind, involvieren Sie den jeweils zuständigen Hintergrunddienst zur Befundinterpretation und Festlegung des weiteren Vorgehens	*–*

Im Folgenden werden nun die einzelnen Schritte des APU-Algorithmus dargestellt. Dabei sind die jeweils genannten SOP entweder als Tabelle in diesem Artikel oder im Onlinezusatzmaterial abrufbar.

### Klinische Einschätzung Schock

Zu Beginn der eEPK erfolgt die klinische Einschätzung bezüglich eines Schocks angelehnt an Tab. 3. Es gilt zu prüfen und frühzeitig zu erkennen, ob eine dringende Indikation zur Notoperation, intensivmedizinischen Verlegung oder unverzüglichen Bildgebung besteht.

Falls mindestens eine dieser Indikationen besteht und ein Patient mit aBS und Schock vorliegt, sollte die unmittelbare fachärztliche/oberärztliche Rücksprache erfolgen und der Algorithmus „Schock bei atraumatischem Bauchschmerz*“ (Feld 9 in *Abb. 1*)* greift. Dieser Algorithmus ist im Bereich der Intensivmedizin verortet und wird hier nicht weiter ausgeführt.

### Basismaßnahmen

Wenn ein Schock ausgeschlossen werden kann, wird der Prozess mit dem Ereignis „*Patient mit Verdacht auf atraumatischen Bauchschmerz ohne Schock liegt vor*“ (Feld 7 in der eEPK) fortgesetzt. Es folgt die Durchführung der Basismaßnahmen. Diese beinhalten die *Anamnese, körperliche Untersuchung *(Feld 10), *Basislabor und die Anlage eines peripheren Venenzugangs *(Feld 14) sowie falls indiziert die *Durchführung eines adäquaten Schmerzmanagements *(Feld 18). Die Vitalparameter sind bereits in der Triage erhoben worden, werden gewürdigt und der Quick-Sequential-Organ-Failure-Assesment(qSOFA)-Score erneut bei erstem Arztkontakt erhoben, um das Sepsisrisiko schnell und einfach abzuschätzen.

Die durchgeführten Basismaßnahmen dienen der Erstversorgung der Patient*innen und sind Grundlage für eine anschließende klinische Einschätzung (siehe Tab. 3). Hierbei soll eine erste Verdachtsdiagnose erarbeitet und dokumentiert werden. Die erneute klinische Einschätzung bietet die Möglichkeit einer Eskalation und zügigen Einleitung des Algorithmus „*Schock bei atraumatischem Bauchschmerz“ *(Feld 9) mit einhergehender ITS- oder OP-Verlegung in Abhängigkeit der Dynamik des Krankheitsbilds.

### Sonographie

Sofern nach der *klinischen Einschätzung* (Feld 23) keine klare Diagnose gestellt werden kann, führt der Prozess entlang der eEPK (Abb. 1) zunächst zur *Durchführung einer Sonographie* (Feld 28) als zentraler diagnostischer Methode, die bettseitig und ohne Strahlenbelastung großzügig indiziert ist [5].

### Fortführende Maßnahmen

Der Patientenpfad gliedert sich nach initialer Sonographie und Postulierung einer Verdachtsdiagnose im Sinne einer interdisziplinären Aufarbeitung weiter auf in erweiterte *radiologische Bildgebung *(Feld 38)*, Observation *(Feld 34) und *Konsil *(Feld 42), sofern nicht direkt nach Sonographie eine eindeutige Diagnose mit nachfolgender spezifischer Therapie und Patientenmanagement möglich ist.

Bei klarem Hinweis auf eine gynäkologische oder urologische Verdachtsdiagnose ist die Notwendigkeit einer interdisziplinären Aufarbeitung in jedem Fall gegeben. Die SOP *Konsil* gibt eine Handlungsübersicht bei entsprechender Verdachtsdiagnose (s. elektronisches Supplement).

Bei dem herausfordernden Symptom aBS sind insbesondere die Akuität und die Dynamik zu berücksichtigen. Do-not-miss-Diagnosen (siehe Tab. 3) müssen bedacht werden. Bei inkonklusiven Befunden stellt die *Observation *(Feld 34) mit erneuter zeitnaher Evaluierung der Befunde unter Einbezug eines interdisziplinären fachärztlichen Teams eine zentrale Rolle des APU-Algorithmus dar. Hierbei ist im zeitlichen Verlauf eine Kontrolle der Vital‑, Laborparameter und Bildgebung anzustreben, um eine Entlassung mit unklarer Diagnose zu verhindern.

Erst wenn eine eindeutige Diagnose aus den vorangegangen Behandlungsschritten vorliegt, führt der Handlungspfad entlang der eEPK zum *spezifischen Management *(Feld 46).

### Spezifisches Management

Das spezifische Management greift, sobald ein Patient mit aBS und eindeutiger Diagnose vorliegt. Als finales Handlungsfeld der APU regelt das *spezifische Management* (Feld 46) den genauen Ablauf der stationären Aufnahme oder Entlassung aus der NA.

Die SOP fokussiert außerdem auf die zentrale Rolle einer frühzeitigen antibiotischen Therapie und der fachärztliche Entscheidung, denen als Qualitätsindikatoren in der NA eine große Bedeutung zukommt.

## Diskussion

Die interdisziplinäre Aufarbeitung der aBS, die Stellung einer Diagnose sowie ein strukturiertes Aufnahme- und Entlassmanagement stellen die wesentlichen Säulen der *Abdominal Pain Unit* dar. Dabei ist „Unit“ nicht als räumlich oder funktionell von der NA getrennter, sondern integrierter, strukturierter Prozess innerhalb der NA zu verstehen. Der Algorithmus der eEPK ermöglicht einen qualitativ einheitlichen Patientenpfad in jeder NA und hat somit das Potenzial, die Versorgungsqualität zu verbessern und eine Reduzierung der Krankenhausmortalität von Patient*innen mit aBS zu erreichen.

Zugleich sollen prozessuale Qualitätsindikatoren implementiert werden, die zukünftig aus Routinedaten ermittelt werden können und damit ein kontinuierliches Qualitätsmanagement bei der Versorgung von Patient*innen mit aBS unterstützen.

In Anlehnung an die CPU mit bereits hoher Bedeutung in der Gesundheitsversorgung, bestehenden Qualitätsindikatoren und bestehender DGK-App soll die APU ebenfalls softwaregestützt den Behandlungsprozess strukturieren und die Behandlungsqualität des Bauchschmerzes sichern. Zur Validierung des Konzepts läuft ab September 2021 eine 2‑jährige deutschlandweite, multizentrische Studie (DRKS-ID: 21052, s. auch apu.charite.de [22]). Bei erfolgreicher Validierung des APU-Prozesses wäre eine feste Integration in die ärztliche Weiterbildung sinnvoll.

Die Notwendigkeit der wesentlichen APU-Prozess-Punkte *Sonographie, radiologische Bildgebung und Konsil* ergibt sich aus den Ergebnisse der CHARITEM-Studie [13].

### Sonographie

In der CHARITEM-Studie [13] zeigte die quantitative Analyse (s. Ergebnisteil), in Einklang mit Ergebnissen aus anderen Kliniken [7], dass die Sonographie in der Diagnostik einen hohen Stellenwert hat. Sie nimmt daher im APU-Prozess eine zentrale Rolle ein und erfolgt stets vor jeder anderen Bildgebung. Die schnelle Verfügbarkeit und sichere Diagnostikmethode bietet die Möglichkeit, akute Krankheitsbilder schnell und ohne Strahlenbelastung zu erkennen[2]. Die Durchführung und Voraussetzungen zur Anwendung sind in der zugehörigen SOP hinterlegt.

### Radiologische Bildgebung

Bei weiter unklarer Diagnose nach Sonographie ist die Computertomographie als zentrale Diagnostikmethode in der Notfallmedizin durchzuführen. Gerade bei über 65-Jährigen liegt die Mortalität beim Leitsymptom Bauchschmerzen bei 5 % [3, 14, 19]. Der diagnostische Wert einer Röntgenübersichtsaufnahme des Abdomens ist limitiert, um relevante, lebensbedrohliche Diagnosen wie freie Luft bei einer Hohlorganperforation auszuschließen [18, 20], und wird sehr oft bei unklaren oder pathologischen Befunden von einer CT gefolgt.

In unserem Patientenkollektiv bei CHARITEM wurde 2010 nur in 3,7 % aller Fälle eine Computertomographie durchgeführt, hierbei wurde in 86,4 % ein pathologischer Befund diagnostiziert. Eine Röntgenübersichtaufnahme des Abdomens wurde in 9,5 % der Fälle eingeleitet, in 49,3 % fand sich bei dieser Diagnostikmethode ein Normalbefund. Der diagnostische Wert dieser Untersuchung ist, wie bei Stoker et al. [18] beschrieben, nicht gegeben. Dies zeigt, dass in dem historischen Patientenkollektiv die CT-Diagnostik noch unterrepräsentiert war und hier keine Schlussfolgerungen zulässt.

### Konsil

Der Abschnitt „Konsil“ hat in der NA eine zentrale Bedeutung. Das Ziel der* Abdominal Pain Unit *ist eine zügige interdisziplinäre Patientenuntersuchung. Die Expert*innenrunde sieht dies, neben einer frühen zweckmäßigen Bildgebung, als essenziellen Punkt einer verbesserten Versorgungsqualität der Patientenklientel mit aBS, um die Krankenhausmortalität zu senken. Die ursprüngliche Fachrichtung der primär behandelnden Person hat auf die Versorgungsqualität keinen Einfluss [9]. Wesentlich ist bei der Notwendigkeit einer fachspezifischen Weiterversorgung, diese rechtzeitig und ggf. durch Verlegung sicherzustellen.

Diese 2 diagnostischen Maßnahmen, Bildgebung und Konsil, sind im Rahmen der eEPK (Abb. 1) als Qualitätsindikatoren, angelehnt an Hörster et al. [6], gut zu erfassen und haben Potenzial, die Versorgungsqualität der aBS in der Notfallmedizin in Zukunft zu sichern.

## Schlussfolgerung

Die hohe intrahospitale Mortalitätsrate sowie die Komplexität des Leitsymptoms aBS verlangen nach einem strukturierten, effizienten und vor allem sicheren Diagnostikpfad im Sinne einer APU. Bei steigenden Patientenzahlen in den Notaufnahmen gilt es, Hochrisikogruppen schnell zu identifizieren. Die APU ermöglicht dies mit frühzeitiger Diagnostik unter Berücksichtigung zeitnah eingesetzter bildgebender Verfahren wie der Sonographie und Computertomographie.

Die Einführung der APU als App-basierten Behandlungspfad wäre ein Fortschritt für die qualitativ einheitliche Versorgung des Bauchschmerzes in jeder NA. Sie hat das Ziel, die Behandlungsqualität zu sichern und die intrahospitale Mortalität langfristig zu senken.

## Fazit für die Praxis

- Die intrahospitale Mortalität von Patient*innen mit akuten Bauchschmerzen ist mit 5,6 %, im Vergleich zum Leitsymptom Brustschmerz (0,9 %), sehr hoch.
- Nicht nur aufgrund des demographischen Wandels, mit steigender älterer Bevölkerung, sondern auch aufgrund des vermehrten Patientenaufkommens in Notaufnahmen müssen die aktuellen Versorgungsstrukturen kritisch evaluiert, standardisiert und effizienter gemacht werden.
- Analog zur „Chest Pain Unit“ wurde ein diagnostischer Behandlungspfad entworfen, der als „Abdominal Pain Unit“ (APU) bezeichnet wird.
- Der Algorithmus der APU ermöglicht einen qualitativ einheitlichen Patientenpfad in jeder NA und hat somit das Potenzial, die Versorgungsqualität zu verbessern und die intrahospitale Mortalität langfristig zu senken.

### Supplementary Information




