# Supplementary material for: The abdominal pain unit as a treatment pathway: Structured care of patients with atraumatic abdominal pain in the emergency department
Source: Med Klin Intensivmed Notfmed. 2021 Dec 20;118(2):132–40. [Article in German] doi: 10.1007/s00063-021-00887-0 (PMC9992050; doi:10.1007/s00063-021-00887-0)
Supplement: Supplementary file 1 [file 63_2021_887_MOESM1_ESM.pdf]

Online-Zusatzmaterial

Standard Operating Procedures der Abdominal Pain Unit

**Tab. S1 Checkliste Anamnese Bauchschmerzen**
Informationstext für App
• Voroperationen<br>• Vorerkrankungen<br>• Schmerzauslöser und Schmerzbeginn<br>• Begleitende Symptome (Sodbrennen, Schluckauf)<br>• Bekannter Schmerz (Vor-Therapien/Befunde)<br>• Schmerzqualität<br>(kolik-/wellenartig, lokalisierbar/diffus, stechend/stumpf, Schmerzbeeinflussung (Verstärkung: Husten, Nahrung, Bewegung, Atmen; Abschwächung: Nahrung, Erbrechen, Stuhlgang, Schonhaltung) Dauerschmerz, Schmerzmaximum, freies Intervall, Schmerzwanderung/Ausstrahlung)<br>• Erbrechen/Übelkeit<br>• Stuhlgang/Miktion (wann zuletzt, Blut, Farbe, Menge)<br>• Gewichtsverlauf<br>• Schwangerschaften/letzte Periode<br>• Letzte Mahlzeit<br>• Auslandsaufenthalt<br>• Medikamentenanamnese

**Tab. S2 SOP Anamnese und körperliche Untersuchung**	
Informationstext aus der App	Items Auswahl in der APP
1. Checkliste Anamnese Bauchschmerz zur Kenntnis nehmen, insbesondere:<br>o Schmerzqualität/-lokalisation/-intensität zur Fokussierung der körperlichen Untersuchung<br><br>2. Körperliche Untersuchung durchführen<br>o Erneute Würdigung der Vitalparameter (HF, RR, AF, SaO2, Temperatur, qSOFA)<br>o Peritonismuszeichen<br>o Blutungszeichen<br>o Ischämiezeichen<br>o Cave besondere Patientengruppen (Schwangere,	**<u>Cor:</u>**<br>☐ rein<br>☐ rhythmisch<br>☐ arrythmisch<br>☐ Systholikum<br>☐ Diastholikum<br>o Punctum maximum<br>▪ Mitrale<br>▪ Aortale<br>▪ Trikuspidale<br>▪ Pulmonale<br>**<u>Pulmo:</u>**<br>Atemgeräusch<br>☐ Vesikulär<br>☐ Bronchial<br>☐ Verschärft<br>☐ Abgeschwächt<br>**<u>Abdomen:</u>**<br>Inspektion:

immunsupprimierte/ältere Patienten)<br>o Alle 4 Quadranten:<br>- Inspektion: prall, marmoriert, Venenzeichnung, Narben, Hernien, Stoma, Drainagen, Katheter<br>- Palpation: bretthart, „Gummibauch“, Loslassschmerz, Abwehrspannung u/o Druckschmerz (lokalisiert/diffus), Leberrand (nach kaudal tastbar, derb, Milz tastbar, Flankenklopfschmerz (einseitig/beidseits), pralle Blase, insgesamt tastbare Resistenzen, Murphy-/Appendizitiszeichen<br>- Perkussion: tympanischer Klopfschall, auslösbarer Peritonismus (diffus/lokalisiert)<br>- Auskultation: Darmgeräusche (DG) vorhanden (regelrecht/rege) oder nicht vorhanden (still), hochklingende DG<br>o Begleitende Allgemeinbefunde: obere Einflussstauung, Leberhautzeichen, Ernährungszustand (adipös/kachektisch), (Skleren-)Ikterus, (Schleimhaut-/Lippen-) Blässe, dünnes Haar, Nageldystrophie, periphere Ödeme<br>o Digital rektale Untersuchung erwägen	☐ Marmoriert<br>☐ Venenzeichnung<br>☐ Narben<br>☐ Hernien<br>☐ Stoma<br>Persistaltik<br>☐ rege<br>☐ spärlich<br>☐ nicht vorhanden<br>☐ hochgestellte Darmgeräusche<br>Palpation weich ☐ hart ☐ Peritonismus ☐<br>Flankenklopfschmerz kein ☐ links ☐ rechts ☐<br>Abwehrspannung<br>☐ li.OQ<br>☐ re.OQ<br>☐ li. UQ<br>☐ re.UQ<br>Druckdolenz<br>☐ li.OQ<br>☐ re.OQ<br>☐ li. UQ<br>☐ re.UQ<br>Perkussion: tympanisch ☐ unauffällig ☐

**Tab. S3 SOP Konsil**	
Informationstext aus der APP	Items Auswahl in der APP
1. Hinweise auf eine **urologische** Ursache der Schmerzen?	☐ Gesicherte Diagnose

- Flankenschmerz/Leistenschmerz/Nierenbeckenklopfschmerz<br>- Dysurie, Algurie<br>- Makro-, Mikrohämaturie<br>- Anamnese, körperliche Untersuchung mit wegweisendem Befund<br>- Sonographisch wegweisender Befund<br>- Bekannte urologische Erkrankung mit Potential zur Akutkomplikation<br><br>Kontaktdaten: Eingabe Klinikspezifisch<br><br>2. Hinweise auf eine **gynäkologische** Ursache der Schmerzen?<br><br>- Bestehende Schwangerschaft, SS-Test durchgeführt? EUG möglich?<br>- Vaginale Blutungen<br>- Anamnese (GV, IUP), körperliche Untersuchung mit wegweisendem Befund<br>- Sonographisch wegweisender Befund<br>- Bekannte gynäkologische Erkrankung mit Potential zur Akutkomplikation<br><br>Kontaktdaten: Eingabe Klinikspezifisch<br><br>3. **Allgemein/Viszeralchirurgische** Vorstellung<br><br>Falls FA Chirurgie noch nicht involviert<br>- akutes Abdomen<br>- Anamnese, körperliche Untersuchung mit wegweisendem Befund<br>- Sonographisch wegweisender Befund<br>- Voroperationen	☐ Unklare Diagnose<br>• Radiologische Bildgebung<br>• Observation

**Tab. S4 SOP Labor bei akuten Bauchschmerzen**	
Informationstext in der APP	Items Auswahl in der APP
1. Venöse Blutabnahme; arterielle Blutgasanalyse bei Hypoxie (SaO2 < 90%)<br>2. Laborparameter (4 Röhrchen + Urin)<br>o Blutbild, maschinelles Differentialblutbild<br>o POC „BGA“ mit pH, Na, K, Laktat, BZ<br>o Chemie: CRP, Lipase, gGT, ALT, Bili, Krea, TSH<br>o Gerinnung: Quick, PTT	☐ Gesicherte Diagnose<br>☐ Unklare Diagnose<br>☐ Schmerzmanagement erledigt<br>☐ Anamnese erhoben

o Urin-Stix<br>Bei Frauen: ß-HCG im Urin	

**Tab. S5 SOP Observation**	
Informationstext aus der APP	Items Auswahl in der APP
**Monitoring**<br><br>**A Zeichen eines Kreislaufschocks sind nicht vorhanden initiale Messung, Wiederholung nach jeweils 30min (*):** RR***,** HF***, je nach klinischem Befund:** Sauerstoff-Sättigung**, 12-Kanal-**EKG-Ableitung; Wiederholung bei Infarktverdacht mindestens nach 3 und 6 Stunden**,** Temperatur (temporal oder rektal; bei Verfügbarkeit am Urindauerkatheter)<br><br>**B Zeichen eines Kreislaufschocks vorhanden:** Durchgehende Monitorüberwachung bis zur Verlegung auf die Intensivstation: RR, HF, Sauerstoffsättigung, EKG-Ableitung<br><br>- **Klinische Visite**<br>o Dokumentation des Verlaufs (Zeitintervall je nach klinischer Ausgangslage explizit planen und dokumentieren):<br>- Symptomatik<br>- klinischer Untersuchungsbefund<br>o Pflegerische Überwachung, regelmäßige Ansprache<br><br>- **Abschließende Wertung** durch den verantwortlichen Facharzt unter Einbeziehung aller erhobenen Untersuchungsbefunde gemäß „SOP Klinische Einschätzung“ und Festlegung des Procedere:<br><br>o **Entlassung**<br>o **Weiterführende Diagnostik (Labor, Bildgebung, Endoskopie)**<br>o **Stationäre Aufnahme**	☐ Entlassung<br>☐ Weiterführende Diagnostik (Labor, Bildgebung, Endoskopie,Konsil)<br>☐ Stationäre Aufnahme<br>☐ Notfall-OP/Endoskopie/Intervention

○ **Notfall-OP/Endoskopie**	

**Tab. S6 SOP Radiologische Bildgebung**	
Informationstext aus der APP	Items Auswahl in der APP
1. Indikationsstellung zur CT-Diagnostik des Abdomens mit KM<br>- Fragestellung<br>- Dringlichkeit<br>2. Check KM-Allergie, Schwangerschaftstest bei gebärfähigen Frauen, TSH und Kreatinin (abhängig von der Dringlichkeit)<br>3. Prüfung alternativer radiologischer Untersuchungen:<br>- Abdomenleeraufnahme in Linksseitenlage und AP im Liegen (nur bei Fragestellung „freie Luft“ oder Ileus „stehende Schlingen“)<br>- in Ausnahmefällen (z.B. schwerer KM-Allergie mit Schock) auch primäres MRT möglich	☐ Gesicherte Diagnose→ spezifisches Management<br>☐ Unklarer Diagnose<br>• Observation<br>• Konsil<br>• OP<br>• Entlassung

**Tab. S7 SOP Schmerzmanagement**	
Informationstext aus der APP	Items Auswahl in der APP
1. Jeder Patient mit akuten oder chronischen Schmerzen erhält ein angemessenes Schmerzmanagement.<br>2. Zu verwendende Skalen zur Schmerzeinschätzung:<br>○ Numerische Rangskala (NRS)<br>○ Visuelle Analogskala (VAS)<br>○ Verbale Rating Skala (VRS) bzw. Begriffsskala<br>3. Patienten mit Sprachbarriere oder eingeschränkter Kommunikationsfähigkeit:<br>○ ALGOPLUS (Einschätzung durch Pflegefachkraft)<br>○ Faces Pain Scale – Revised (FPS-R)	Schmerzen ☐akut ☐chronisch<br>Schmerzskala NRS<br>0__1__2__3__4__5__6__7__8__9__10<br><br>VAS<br>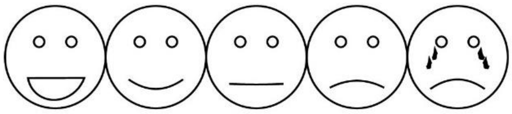<br><br>vor Medikamentengabe:<br>☐ Schwangerschaft/Stillzeit<br>☐ Allergien<br>☐ Porphyrie<br>☐ schwere Leber- und Nierenerkrankungen<br>☐ Epilepsie/neurologische Erkrankungen

4. Erhebung vor jeder Medikamentenverabreichung:<br>o Schwangerschaft/Stillzeit<br>o Allergien<br>o Porphyrie<br>o schwere Leber- und Nierenerkrankungen<br>o Epilepsie/neurologische Erkrankungen<br>o Vitalparametern<br>5. Bei akuten Schmerzen und fehlenden Kontraindikationen ist die Verabreichung von Analgetika auch in Rücksprache mit dem AvD vor Arztkontakt möglich.<br>6. Gleiches gilt bei fehlenden Kontraindikationen für (Dosierung siehe Schmerzschema*):<br>o Antiemetika*<br>o Spasmolytika*<br>o PPI*<br>7. Nach 30 Minuten sollte eine erneute Abfrage anhand der Schmerzskala durchgeführt und dokumentiert werden (von Pflegkraft oder Arzt)<br>8. Bei anhaltenden Schmerzen, Verschlechterung des Allgemeinzustandes ist sofort ein Arzt hinzuzuziehen.<br>9. Die Abgabe von Opioiden* erfolgt ausschließlich nach Arztkontakt (Dosierung siehe Schmerzschema*).<br>10. Die entsprechende Dokumentation muss in der BTM-Dokumentation und im EH-Schein erfolgen.<br><br>**Schmerzschema Medikamentenoptionen**<br>Analgetika:<br>o Novaminlsulfon 1,0 g oder 2,5 (Cave RR) i.v. als Kurzinfusion; Maximaldosis: 5.0 g /d	☐ Vitalparametern<br><br>Medikament erhalten<br>☐ ja<br>☐ nein

○ Paracetmol 1 g i.v. als Kurzinfusion bis 4×/Tag, Maximaldosis Paracetamol 4,0 g/d<br>○ Opioide: Piritramid (7,5 mg i.v. als Kurzinfusion über 15 min); Maximaldosis: 30 mg/d<br>PPI:<br>○ Pantoprazol 40 mg p.o. oder i.v.<br><br>Spasmolytika:<br>○ Butylscopolamin 20mg i.v./p.o. 3-5×/Tag, Maximaldosis 100mg/d<br><br>Antiemetika:<br>○ Metoclopramid 10 mg p.o / i.v. , Maximaldosis 30mg/d<br>○ Ondansetron 4mg s.l. oder bei Chemotherapie Bolus: 8 mg i.v. als Kurzinfusion über 15 min, Maximaldosis 32 mg/d<br><br>In der Schwangerschaft bevorzugt:<br>○ Paracetamol<br>○ Buscopan<br>○ Omeprazol 20 - 40mg po	

**Tab. S8 SOP Sonographie**	
Informationstext aus der AP	Items Auswahl in der APP
○ Durchführung der Sonographie mit SIN Level-II<br>○ Point-of-Care-Ultraschall Akutes Abdomen - fokussierte Abdomensonographie<br>- Aszites / freie Flüssigkeit<br>- Ileuszeichen / pathologische Darmdistension, Pendelperistaltik, großer Flüssigkeitsgefüllter Magen<br>- Intra-/extrahepatische Cholestase/ Cholezystitis<br>- Pankreatitis	☐ Gesicherte Diagnose<br>☐ Unklarer Diagnose

- Nierenversagen / Harnstau / Urolithiasis/ Harnverhalt<br>- Beurteilung Leber (Abszess, RF)<br>- Beurteilung Milz (Abszess, Einblutung, Ruptur)<br>- Appendizitis (Kokardenphänomen, Wandverdickung, Abszess)<br>- Sigmadivertikulitis (entzündliche Wandverdickung +/- Hyperämie, Darstellbare Divertikel, Abszess)<br>o Digitale Bilddokumentation<br>o Besprechung der Bilder mit Oberarzt/Facharzt (Innere Medizin/Radiologie/Allgemeinchirurgie) mit spezieller Erfahrung Notfallsonographie<br>o Verfassen des Befundberichtes<br>o Bei Veränderung des AZ / Verdachtsdiagnose evaluiere Kontroll-Sonographie und Indikation zur Durchführung einer zeitnahen CT-Untersuchung (< 2h)	

**Tab. S9 SOP Spezifisches Management**	
Informationstext aus der APP	Items in der APP
0. Schmerzmanagement re-evaluieren! → SOP Schmerzmanagement<br>1. Rücksprache Facharzt, falls bisher nicht erfolgt<br>2. Entlassung aus Notaufnahme geplant:<br>o Überprüfe APU-Algorithmus auf Vollständigkeit von<br>- Anamnese<br>- Körperlicher Untersuchung<br>- Diagnostik<br>- Therapie<br>o Überprüfe Vollständigkeit der Entlassungsdokumentation<br>o Entlassungsgespräch führen<br>o Wiedervorstellung (ambulant/stationär) evaluieren und Termin vereinbaren<br>o Entlassung ausschließlich nach Rücksprache mit dem dazu im Klinik Kontext befugten Facharzt („Hintergrund“)	<u>Schmerz</u><br>Schmerzskala NRS<br>0__1__2__3__4__5__6__7__8__9__10<br><br>VAS 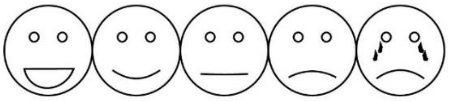<br><br><u>Entlassung</u><br>**Nein** ☐<br>o Observation in Notaufnahme<br>o Stationäre Aufnahme<br>o Pat. Informiert<br>o Bettenmanagement/Station informiert<br>o OP/Intervention<br>o Aufklärung<br>o Post-Op Versorgung geklärt<br>**Ja** ☐

3. Antibiotische Therapie indiziert? Weitere spezifische medikamentöse Therapie?<br>o Akute Cholezystitis, akute Appendizitis, akute Divertikulitis, inkarzerierte Hernien<br>o Bei Zeichen der Infektion/Sepsis auch, wenn Fokus nicht sicher!<br>o Weitere spezifische medikamentöse Therapie mit Hintergrunddienst klären!<br>4. Stationäre Aufnahme geplant:<br>o Patientengespräch<br>o Level of care festlegen<br>o Frühzeitige Info an Bettenmanagement (lokale Regelungen)<br>o Info ad Station, Anordnungen festlegen, um Lücken zu vermeiden (Kreislaufüberwachung? Thormboseprophylaxe? Medikation? Kost?)<br>o Andere Berufsgruppen (insbesondere Pflege) einbeziehen und informieren<br>5. Direktverlegung in den OP oder Endoskopie geplant:<br>o Patientengespräch, Aufklärungsgespräch<br>o Einwilligung einholen (lokal übliche Bögen)<br>o Info an den OP-Koordinator/Endoskopieanmeldung, ggf. Team alarmieren<br>o Bei OP: Lokale Checkliste "OP-Vorbereitung" abarbeiten	Überprüfe APU-Algorithmus auf Vollständigkeit von<br>o Anamnese<br>o Körperlicher Untersuchung<br>o Diagnostik<br>o Therapie<br>o Überprüfe Vollständigkeit der Entlassungsdokumentation<br>o Entlassungsgespräch führen<br>o Wiedervorstellung (ambulant/stationär) evaluieren und Termin vereinbaren<br>o Entlassung ausschließlich nach Rücksprache mit dem dazu im Klinik Kontext befugten Facharzt („Hintergrund“)<br><br>Antibiotikagabe nötig? ja ☐ nein ☐<br>Behandlungsende: Datum/Uhrzeit der Anmeldung der Entlassung/Verlegung
